# Mobilizing knowledge about urban change for equity and sustainability: developing ‘Change Stories’, a multi-country transdisciplinary study

**DOI:** 10.12688/wellcomeopenres.21180.1

**Published:** 2024-04-24

**Authors:** Helen Pineo, María José Álvarez Rivadulla, Elis Borde, Waleska Teixeira Caiaffa, Vafa Dianati, Geraint Ellis, Friederike Fleischer, Adriana Hurtado Tarazona, Olga L. Sarmiento, Agustina Martire, Sergio Montero, Gemma Moore, Rebecca Morley, Aarathi Prasad

**Affiliations:** 1 Department of Urban Design and Planning, University of Washington, Seattle, Washington, 98105, USA; 2 Institute for Environmental Design and Engineering, Bartlett School of Environment, Energy and Resources, University College London, London, England, WC1H 0NN, UK; 3 Sociology, School of Social Sciences, Universidad de los Andes, Bogotá, Colombia; 4 Department of Preventive and Social Medicine, School of Medicine, Federal University of Minas Gerais, Belo Horizonte, Brazil; 5 Development Planning Unit, University College London, London, England, WC1H 9EZ, UK; 6 School of Natural and Built Environment, Queen's University Belfast, Belfast, Northern Ireland, BT9 5AG, UK; 7 Department of Anthropology, Universidad de los Andes, Bogotá, Colombia; 8 Interdisciplinary Center for Development Studies (CIDER), Universidad de los Andes, Bogotá, Colombia; 9 School of Medicine, Universidad de los Andes, Bogotá, Colombia; 10 Department of Human Geography, University of Toronto, Scarborough, Toronto, Ontario, Canada; 11 Rebecca Morley Consulting, Wilmington, Delaware, 19801, USA; 12 UCL Research Department of Genetics, Evolution and Environment, University College London, London, England, UK

**Keywords:** Health, Equity, Transdisciplinary, Decolonial, Methods, Participatory, Narrative, Urban, Transformation

## Abstract

**Background:**

Health-focused research funders increasingly support multi-country research partnerships that study health, urban development and equity in global settings. To develop new knowledge that benefits society, these grants require researchers to integrate diverse knowledges and data, and to manage research-related aspects of coloniality, such as power imbalances and epistemic injustices. We conducted research to develop a transdisciplinary study proposal with partners in multiple middle and high income countries, aiming to embed equity into the methodology and funding model.

**Methods:**

Parallel to literature review, we used participatory and social research methods to identify case study cities for our primary study and to inform our study design. We conducted semi-structured interviews with informed and consented sustainable urban development experts in the USA (n=23). We co-developed our research approach with our global advisory group (n=14) and conducted a participatory workshop (n=30) to identify case study sites, also informed by conversations with international academic experts in sustainable development (n=27).

**Results:**

Through literature review we found that there is a need to study the contextual pre-conditions of urban transformation, the influence of coloniality on understandings of how cities can change and the failure of standard development practices to meet the needs of all residents and the planet. Through expert input and literature we found that decolonial and storytelling methods may help us show the complexities behind stories of urban transformation, particularly the role of marginalized populations in creating long-term change.

**Conclusions:**

There are multiple benefits of conducting research to develop an equitably designed multi-country research collaboration. We built new partnerships and co-developed our research approach, creating new understanding of diverse collaborators’ disciplinary perspectives and institutional requirements. By investigating the informational needs of U.S. sustainable development actors and designing our study to meet these needs, we have increased the likelihood that our research will create impact.

## Plain language summary

Project title: Mobilizing knowledge about urban change for equity and sustainability: developing ‘Change Stories’, a multi-country transdisciplinary study. Our team initially included researchers in London and later expanded to include international collaborators with expertise in urban development, public health and anthropology. This project was primarily conducted through online workshops and interviews (May – Dec 2022), with one in-person workshop in Valencia, Spain (Oct 2022). Research funders have recently increased support for multi-country partnerships that study urban health equity. However, there is often a lack of funding to develop these studies in a way that supports integration of diverse fields and manages research-related aspects of coloniality, such as power imbalances and devaluing certain forms of knowledge. We conducted research to develop a transdisciplinary study proposal with partners in multiple countries, aiming to embed equity into our approach. We used literature review alongside social research methods (94 participants) to develop case study cities and our study design. We found that community involvement in urban governance is important for achieving equity and health, yet it remains under-valued in status quo development models, undermining the potential for cities to support people and the planet. We believe that our approach offers valuable learning for other multi-country transdisciplinary studies.

## 1. Introduction

A lack of progress on just urban development has prompted recent investigations of processes and policies that promote equity ( Castán Broto *et al.*, 2022; Dsouza *et al.*, 2023). Health-focused research funders have recently increased support for multi-country research partnerships working at the intersections of health, urban development and equity, such as the Wellcome Trust’s Our Planet Our Health grants ( Diez Roux *et al.*, 2019; Pineo *et al.*, 2021a). To develop new knowledge that benefits society, these transdisciplinary grants should confront research-related aspects of coloniality, such as power imbalances and epistemic injustices that have characterized global health research, among other fields ( Hodson *et al.*, 2023). This paper describes the results of research conducted to develop the design, partnerships and funding model for a multi-country transdisciplinary study which embeds equity principles ( ESSENCE on Health Research and UKCDR, 2022). The study is called ‘Change Stories: Learning from narratives of equitable and sustainable urban transformation’. 

*Change Stories* aims to learn from cities that have achieved transformational change toward urban equity and sustainability. We consider transformational urban change to most likely result from actions that target multiple outcomes (e.g. health and climate adaptation), achieved by multiple actors operating at different scales (i.e. micro, meso, and macro). We also agree with Künkel and Ragnarsdottir’s ( 2022, p. 2) definition that emphasizes social change, where transformation is ‘the planned change endeavors that involve deeply innovative approaches towards thinking and acting, and that question as well as shift power structures and relationships’. There is considerable debate about definitions of transformational change ( Crane *et al.*, 2021) and there are concerns that examples of transformative planning, such as Barcelona’s Superblocks, have inadequately addressed equity ( Anguelovski *et al.*, 2023). In a substantial shift over the last 30 years, the United Nations’ now places cities as the governance tier leading innovations to reduce inequalities and support planetary health ( Parnell, 2016). The Sustainable Development Goals (SDGs) aim to combat inequalities, yet they contain contradictions and ‘noticeable silences’ on key drivers of urban inequity ( Butcher *et al.*, 2021). From a public health perspective, environmentally sustainable urban development clearly supports health (and other) co-benefits ( Howden-Chapman *et al.*, 2015; de Oliveira *et al.*, 2015), but without explicit attention to equity, such development will not necessarily improve health for those with the greatest need.

Despite attention from the SDGs, equity remains a poorly understood pillar of sustainable development and inequities continue to grow within cities and internationally ( Álvarez-Rivadulla *et al.*, 2019). Achieving urban equity requires confronting status quo distributions of power and resources and thus it remains one of the most challenging aspects of sustainable development in conventional political and policy processes. Julian Agyeman ( 2005) called this lack of progress the ‘equity deficit’ and it became the impetus for his reconceptualization of sustainability, clarifying *what* is being sustained, *by* whom, *for* whom and *how*. Agyeman’s ( 2013) definition of ‘just sustainabilities’ acknowledges ‘the relative, culturally and place-bound nature of the concept’ (thus the plural form) offered as an alternative to the widely used Brundtland definition ( WCED, 1987). The concept of ‘just sustainabilities’ integrates four equal conditions: 1) improving quality of life and wellbeing, 2) meeting the needs of current and future generations, 3) producing justice and equity through recognition (process, procedure and outcome) and 4) living within the limits of our planet ( Agyeman, 2005; Agyeman, 2013). Although there has been slow progress on integrating equity into urban development ( Castán Broto & Westman, 2017), in some countries, the COVID-19 pandemic and concurrent increased awareness of social movements, such as Black Lives Matter, may mark a turning point in how equity is understood and valued by urban leaders.

Public policy responses to COVID-19 also exposed the limits of rational evidence-based policy models among the health research community, drawing attention to the need for research on the complex and deeply contextual policy processes that underpin successful change. Scientific evidence is only part of policy-making ( Cairney & Oliver, 2017), a process that is heavily constrained by deep political divisions about the problems of our time – climate, growing intersectional inequities and structural racism being paramount. Evidence-based solutions to these problems are not lacking ( Gelderloos, 2022; Lomborg, 2007); instead, we lack agreement about the fundamental nature of the issues facing urban communities and how cities could solve them through physical and social transformations. Even where there is agreement on the nature of the problem, current ways of documenting and sharing successful examples of sustainable development are not necessarily sufficient for replication because the knowledge about the transformation and the actions driving it are deeply context-specific ( Hölscher & Frantzeskaki, 2021). In this context, the Robert Wood Johnson Foundation supported a research project to learn from the cultures, narratives and mindsets that have succeeded in dramatically shifting the status quo of urban development, toward equity, sustainability and health. Although the results are primarily intended to benefit U.S. audiences, the Foundation encouraged us to develop an equitable and non-extractive research design and approach within the development grant for *Change Stories* (the focus of this paper).

The project development phase of transdisciplinary research is often under-funded ( Pineo *et al.*, 2021b) and through this paper we aim to demonstrate both the challenges associated with developing such studies and the value of providing researchers with the necessary time and resources to scope methodologies and build partnerships. This paper reports our activities and findings from the *Change Stories* development grant, comprised of the following objectives: 1) determine sustainable and equitable urban development knowledge gaps in U.S. cities; 2) co-produce a conceptual approach and project structure for working across diverse geographies; and 3) collaboratively select sites and partners to study narratives of equity-driven sustainable urban development. The originality of this paper is that we open the black box of multi-country transdisciplinary study design, describing how we navigated diverse theories and conceptualizations of our research problem to develop a study design, funding model and partnership structure. In reporting this research, we aim to improve funders and researchers’ understanding of the resources and activities required to fully conceptualize multi-country studies that transcend conventional academic structures and boundaries. The next section describes the broad challenges and key tenets of our research and positions this study in the fields of sustainable development, urban transformation, storytelling and decolonial approaches to urban health studies. We then describe our overall research approach, which is reflexive and experimental, building on a model for transdisciplinary research ( Pineo *et al.*, 2021b). The following section describes findings of expert interviews and literature about the information needs of U.S. practitioners regarding sustainable and equitable urban development. A parallel process of case study selection is then reported, describing the results of an expert-informed and participatory process. We then outline our approach for the full *Change Stories* study, including a description of our collaboration principles and research plan. We close with a brief discussion of the strengths and limitations of our approach, including reflections on the impact of this development grant on the wider risks and benefits of multi-country transdisciplinary projects.

## 2. Mobilizing knowledge of equitable urban change

Within our study’s aim of learning from successful experiences in diverse cities, our early project conceptualization identified a number of challenges that would influence both the subject and methodology of our study. Principally, few cities have surpassed standard development practices toward healthy, equitable and sustainable models, and where this has occurred, the full story has not been told. In addition, there are three broad challenges related to knowledge and action for sustainable development germane to the research focus, and described in greater detail below: 1) urban transformation research has largely neglected to study the complex contextual pre-conditions of change, leading to a flawed understanding of how change could occur elsewhere; 2) coloniality influences conceptions of urban problems and solutions, inhibiting the generation of contextually appropriate research and policy approaches; and 3) conventional development practices fail to meet the needs of all residents and the planet, resulting in negative health, wellbeing and environmental impacts. This section positions our study within current theory and understanding of these challenges.

### 2.1. Contextual knowledge of urban transformation

Many disciplines have approached the question of how cities change and specifically how they can be directed toward non-linear sustainability transitions that support health ( Crane *et al.*, 2021). By non-linear, we reference a frequent component of transformation literature, that change must be rapid and at a large scale, not incremental. There is a lack of consensus on how to define and achieve transformational change (ibid). In contrast to the view that transformation can only be achieved through interventions with speed and large scale, an alternative is that incremental improvements from bottom-up and top-down initiatives could lead to transformative urban changes, particularly if public awareness, industry norms and political agendas shift in response to such micro changes ( Pineo, 2022). Conceptualizations of transformation in the sustainability transitions literature respond to the existential crisis of climate change and largely seek to create change within existing political economic and governance structures. Others critique the systems that led to society’s deepest challenges, urging us to view transformation as necessitating more fundamental changes in resource distribution, power and knowledge ( Künkel & Ragnarsdottir, 2022; Suppiah *et al.*, 2022). 

To make sense of the many theories and fields contributing to the urban transformation topic, Hölscher and Frantzeskaki ( 2021) categorized three strands of research in this area, providing an indication of their respective benefits and disadvantages. First, transformation *in* cities represents approaches that explore ‘the diverse factors, processes and dynamics driving place-based transformations’ and ‘explain why transformations occur and are supported in some places and not others’ (ibid, p.1). Experimental governance approaches and experimentation with urban solutions (e.g. demonstration projects) are characteristic of this approach ( Bulkeley *et al.*, 2019), which can be scaled up through processes of social learning ( Sengers *et al.*, 2019). Hölscher and Frantzeskaki note that this perspective may limit transferability of successful sustainable development approaches because the knowledge about the transformation and the actions driving it are deeply context specific.

A second strand of research on urban change is summarized as transformation *of* cities, which studies ‘the outcomes of transformative changes in urban (sub-)systems’ and seeks to ‘evaluate the emergence of new urban functions, new interactions and their implications for sustainability and resilience’ ( Hölscher & Frantzeskaki, 2021, p. 1). This approach views transformation as arising from adjustments to dynamic urban systems. By defining goal states, scholars seek to identify synergies and trade-offs of specific actions within city (sub-)systems ( Elmqvist *et al.*, 2019; McPhearson, 2020). A limitation of this perspective is its underappreciation of local contestations, politics, conflict and place-specific implications ( Hölscher & Frantzeskaki, 2021).

Finally, the transformation *by* cities research strand addresses the global and regional effects of urbanization and urban development. Cities are ‘changing the world’ and should be ‘partners in global governance’ ( Acuto, 2016). Through investigation of the diffusion of best practices ( Lam *et al.*, 2020), scholars aim to ‘inform and reinforce global agendas for action’ ( Hölscher & Frantzeskaki, 2021). A risk with this perspective is its focus on leading global cities at the expense of smaller and less economically dominant cities (ibid). Hölscher and Frantzeskaki argue that this research strand would be improved through a more contextual research approach and ‘comparative analyses into the factors and dynamics influencing place-based transformations’ (p.9). Geography scholars, such as Robinson ( 2006, 2016, 2018), have raised attention to the challenges of urban comparative research, while highlighting its potential if done with consideration of what McFarlane ( 2010) terms ‘theory culture, learning, and ethico-politics’. Along these lines, Montero and Baiocchi’s (2022) ‘a posteriori comparisons’ methodology offers a promising approach that emphasizes comparing urban processes (and ‘repeated instances’ of these) rather than specific places or types of cities, and the production of mid-level theory in place of universal or overly narrow interpretation.

Alongside categorization of how urban transformation is studied and understood, there has also been considerable research identifying specific conditions or capacities underpinning urban change. Wolfram’s ( 2016) review of seven research strands that address urban transformation highlights their tendency to focus on technological and resource capacities, rather than the ‘intangible structures and practices’ that are ‘critical preconditions’ for transformative change, ‘including stakeholder autonomy and empowerment, collaborative governance, and transformative leadership’ (p.129). From this review, he developed a framework of 10 interdependent components for urban transformative capacity ( Table 1), defined as

‘the collective ability of the stakeholders involved in urban development to conceive of, prepare for, initiate and perform path-deviant change towards sustainability within and across multiple complex systems that constitute the cities they relate to. It is a qualitative measure for an emergent property that reflects attributes of urban stakeholders, their interactions, and the context they are embedded in’ (p.126).

**Table 1.** Components of Wolfram's ( 2016, pp. 127–128) conceptual framework for urban transformative capacity.

No.	Component
1	Inclusive and multiform urban governance
2	Transformative leadership (in the public, private and civil society sectors)
3	Empowered and autonomous communities of practice (place-based and/or issue-driven)
4	System(s) awareness and memory
5	Urban sustainability foresight
6	Diverse community-based experimentation with disruptive solutions
7	Innovation embedding and coupling
8	Reflexivity and social learning
9	Working across human agency levels
10	Working across political-administrative levels and geographical scales

Equity is present in his framework in the ‘empowered and autonomous communities of practice’ component (i.e. to analyze and meet social needs, prioritizing deficits in policy) and the ‘inclusive and multiform urban governance’ component (i.e. including formerly excluded stakeholders, procedural equity) ( Table 1). Castán Broto and colleagues ( 2019) looked for the adoption of components in Wolfram’s framework in 400 sustainability initiatives. They found that few initiatives demonstrated transformative capacity dimensions. The most widely satisfied components were participation/inclusiveness and meeting social needs, but these were only found in 27% and 35% of initiatives (respectively). This evidence highlights that capacities to produce both transformation and equity through urban development are lacking.

Both Wolfram (2016) and Castán Broto *et al.* (2019) emphasize the importance of social learning and reflexive action as a part of transformative capacity that may help unlock other components of Wolfram’s framework. Learning and reflexivity are central to studies of sustainability transition as means of evaluating whether a specific initiative is working (and making improvements where needed, so-called first order learning), to change actors’ understanding of the types of activities and processes that function to produce desired outcomes (second order learning) and to inform cross-case learning ( Luederitz *et al.*, 2017). Studying examples of urban transformation should therefore include examination of the types of learning and reflexivity that have occurred. These activities may be informal, not framed as technical models of evaluation, and likewise may occur as part of successful examples of ‘sustainable’ and ‘equitable’ urban change that are not labelled using those terms.

This brief overview of urban transformation research highlights that this area of study has largely neglected the complex contextual pre-conditions of change in cities, leading to a flawed understanding of how change could occur elsewhere. The *Change Stories* project can benefit from existing urban transformation literature; however, there is a need for a deeper investigation of the context, narratives and cultures from multiple actors that led to change. We believe that the dominant narrative of change (how it occurred and who was responsible) in a particular city may not match residents’ experiences, creating a dissonance ( Phelps & Valler, 2018) that requires exploration in order to learn from urban change examples. Our efforts to learn across cities will benefit from post-colonial contributions to comparative urban research and policy mobilities, specifically Montero and Baiocchi’s (2021) ‘a posteriori comparisons’ (described in Section 6).

### 2.2. Coloniality and knowledge of cities

Coloniality is at the heart of the hierarchization of knowledge and narratives about cities, shaping what knowledge is considered to be legitimate to inform urban decision-making at many levels and which models of urban development are considered acceptable and superior. By coloniality, we mean what Mignolo ( 2012, p. 25) defines as ‘a matrix for management and control of the economy, authority, knowledge, gender, sexuality, and subjectivity’. It is not only a question of a colonial model of urban planning and governance, rather there is a fundamental need to critically evaluate the ontologies and epistemologies that inform how we understand cities and urban change. Existing theories and conceptualizations of sustainable and equitable urban development are influenced by coloniality, from the definition of key terms to the theoretical lenses through which researchers interpret data ( Kothari *et al.*, 2019). Through this critical lens, we identify multiple ways in which knowledge about cities are influenced by coloniality.

Non-extractive research approaches sharply contrast with academic extractivism, challenging the prevalent practices of resource extraction from research 'fields' and 'subjects' in the global South by academia in the global North. Academic extractivism as an exploitative pursuit and primarily motivated by the quest for research project development and funding, disrupts the academic landscape, prioritizing quantifiable, short-term gains at the expense of enduring impacts on disadvantaged communities. Scholars such as Cusicanqui ( 2012) characterize this as a form of 'conditional inclusion,' where subaltern and indigenous subjects are reduced to mere sources of data. In this process of value extraction, knowledge is decontextualized, and its legitimacy is contingent upon how the North represents it ( Cruz & Luke, 2020; Grosfoguel, 2007; Simpson, 2014). The resulting knowledge disproportionately benefits the North, sustaining cycles of underrepresentation and exploitation in subaltern/indigenous contexts, also leading to the discreet commodification of poverty in the global South and the accumulation of cultural capital in the North. Epistemic extractivism inadvertently simplifies the multifaceted experiences of marginalized populations into narratives that reinforce stereotypes, perpetuating social inequalities. This approach poses ethical dilemmas by marginalizing the voices and knowledge of disadvantaged communities, fostering exploitative research endeavors that paradoxically fetishize their struggles.

Standard professional practices for how urban changes are imagined and should be managed are influenced by coloniality and have resulted in harms to people and the planet. Watson ( 2009) observed that many urban planning and development practices in the global South were inherited through colonialism or ‘adopted from Northern contexts to suit particular local political and ideological ends’ that resulted in harms to low-income populations. In the U.S., Barry and Agyeman ( 2020) elucidate the impact of coloniality on the marginalization of urban indigenous populations. Globally, there is evidence of the modern impact of colonial planning models which continue to create and reinforce social and spatial inequities ( Kothari *et al.*, 2019; Porter, 2010). Colonialism in the U.S. has not only harmed indigenous populations, but through its pillars of racism and capitalism it has created numerous environmental, economic and social challenges exacerbated by inequitable distribution of resources. The historical practice of redlining in the 1940s meant that neighborhoods inhabited by low-income and Black, Indigenous and People of Colour (BIPOC) populations did not have the same access to quality infrastructure as neighborhoods populated by white and wealthier people, such as schools, housing and services. Research has evidenced multiple negative effects of redlining for health and quality of life, such as greater exposure to overheating and air pollution and reduced life expectancy in redlined areas ( Hoffman *et al.*, 2020; Hsu *et al.*, 2021; Huang & Sehgal, 2022; Lane *et al.*, 2022). Contemporary climate adaptation projects may continue to reinforce hierarchies and privileges determined by coloniality, including through green infrastructure planning ( Shokry *et al.*, 2023).

Cities face many common challenges around the world, but systems for transferring learning between cities have tended to prioritize European and global North cities as leaders, delegitimizing and reducing the potential for other learning ( Healey, 2013; Myers, 2014; Robinson, 2018). The global movement of policy ideas has reinforced adoption of urban development models that are top-down and benefit wealthy residents while producing unhealthy living circumstances for marginalized groups. Furthermore, in those cases in which we learn from the South, successful examples of urban change, known as ‘best practices’, often focus on a simplified account that leaves out the full story (or stories), obscuring our knowledge of how urban transformations occur and hampering our ability to recreate successes internationally ( Montero, 2017; Montero, 2020). Urban policy elites, multinational companies and international development organizations have often resorted to storytelling to produce and mobilize certain policy models ( Montero & Baiocchi, 2021), to circulate corporate discourses around smart cities ( Söderström *et al.*, 2020) or to brand cities to particular audiences ( Duque Franco & Ortiz, 2020). Whose stories are told and whose are ignored in dialogues about urban futures determines the perspectives and priorities that are given weight in decision-making and therefore who benefits, reinforcing existing inequities.

Given that coloniality is deeply intertwined with existing urban form and governance, and our conceptualizations of how cities should change in the face of 21 ^st^ century challenges, it is clear that achieving equitable development requires discarding or working around conventional development models. Undoing coloniality’s harmful impacts requires fundamental shifts in how we identify and value different types of knowledge. Mignolo ( 2012) refers to this task as decolonizing epistemology, starting with questioning and disengaging from ‘the principles of knowledge and understanding that regulate Western society, its imperial expansion and its adaptations in non-European nations’ (p.23). In turn, this requires practices of ‘learning to unlearn in order to relearn’ and to ‘dwell and think in the border’ (p.26). The next section describes how stories and practices of storytelling have been advocated as mechanisms to decolonize research and planning; they also create new possibilities for urban futures.

### 2.3. Narratives and stories of urban change

Stories and narratives are pivotal in current debates about equitable urban development because they are seen as powerful motivators of change and constitutive of our urban future. Forms of storytelling are not only seen as a central part of planning and policy practice ( Hoch, 1994; Innes, 2004; Jones *et al.*, 2014; Sandercock, 2003; Throgmorton, 1996), but they are given great weight in their potential to be a catalyst for urban change ( Sandercock, 2003; Sandercock, 2010) and ‘the linchpin for decolonizing planning’ ( Ortiz, 2022). Harris ( 2023) states that ‘the true power of stories’ is in creating possibility to navigate crises, as they ‘invite us to sit with, and deeply feel, both complexity and possibility’. Fisher’s ( 1989) theory of narrative paradigm confers a far greater power to stories and narrative, arguing that they are the basis through which we experience and comprehend life, including how we form and share our beliefs and motivation for action. In this vein, narratives as ‘patterns in stories’ ( FrameWorks Institute, 2021) can be leveraged to change mindsets, leading to social change ( FrameWorks Institute, 2020). Indeed, policy narratives, or stories ‘told to sway opinion’ are used by politicians and social movements to create policy change, even (or especially) when facts are at odds with the chosen narrative ( McBeth *et al.*, 2022). A recent example was the ‘Yellow Vest’ movement in France that used a narrative about economic harm to the working class (portrayed as heroes) to resist climate mitigation policies imposed by urban politicians (portrayed as villains) (ibid). Despite the important role of narrative in urban policy-making and the long history of people telling stories as a catalyst for change ( Sandercock, 2003), the opportunity to learn from and leverage stories has been under-represented in research about urban transformation.

Ortiz ( 2022) calls for a form of storytelling that can lead to transformative change by activating agency and disrupting existing ways of thinking and power. Ortiz describes ‘storytelling otherwise’ as a practice for decolonizing planning that can support epistemological justice and healing. Storytelling otherwise is exemplified in Ortiz’s paper by the story of a woman called Mama Chila with extracts used to illustrate the four underpinning concepts from Latin American decolonial thinking. First, *epistemological disobedience* involves ‘cultivating an emphasis on the experiences of the knower (enunciation) rather than the known (enunciated) and the relationship between the two’ (ibid, p.14). It prioritizes collective stories over individual accounts, the latter being characteristic of ‘neoliberalism’s emphasis on hyperindividualism’ that feeds current crises of isolation and loneliness ( Harris, 2023). Second, *pluriverse imagination* challenges the universality of knowledge claims and the dominance of extractive growth models. Ortiz ( 2022) explains that this approach requires us to ‘centre stories of transformative experiences of various genealogies prefaced on autonomy and conviviality such as degrowth, buen vivir, ubuntu, eco-feminism and so on’ (p.15). Third, the concept of *sentipensante* or ‘to think and feel with the territory using ancestral knowledges, collective affection and people’s economies’ can support the overcoming of ‘individualistic, dualistic, logocentric and anthropocentric approaches of storytelling in planning’ (p.15). Emotions are part of reason in this approach.

A final benefit that Ortiz offers from ‘storytelling otherwise’ is its capacity to bring about ‘ *border thinking for communal healing*’, an ongoing process rather than a final state, similar to the wider process of decolonization (p.16–17). Border thinking refers to the threshold and dialogue between diverse knowledge systems. Ortiz references the need for healing from collective traumas inflicted by violence, including those brought about by urban development. Mama Chila’s story speaks of pain from many sources including an ‘upgrading macroproject’ that left her family with a ‘big land tax debt’ meaning that they lost their house and struggled to stay in the community that they had built. Sharing Mama Chila’s story displaces the dominant narrative that residents’ lives will be improved through top-down transformative change, while highlighting alternative forms of urban change.

As a central way that people understand themselves and their culture, storytelling is advocated as a methodology for broadening the voices and knowledges that shape scientific understanding in an increasing number of disciplines ( Coombes *et al.*, 2014; de Leeuw *et al.*, 2017; Harris, 2023; McCall *et al.*, 2021). Storytelling as a research methodology and method has specific benefits for participants and researchers distinct from other approaches. In relation to health, storytelling methods may produce more ‘nuanced information and emotions’ and more ‘subtle insights on behavior’ than other methods ( McCall *et al.*, 2021). De Leeuw *et al.* (2017) critically evaluated the benefits and risks of storytelling in geography and medical-health sciences. Reported benefits included its power to create rich data about culture and context, to be a safe way to discuss difficult experiences or issues (non-hierarchical and non-threatening) and to connect to wider audiences. The risks with storytelling are partly about ethics, being ‘attentive to not steal the stories’, and partly about stories being co-opted and losing their power (ibid, p.157). They write that researchers must be ‘sufficiently vigilant and critically aware to ensure [stories] do not become a parody of themselves, something wholly corruptible and able to be put to use in exactly the opposite ways as those for which they were intended’ (ibid). In addition to critical self-reflection, de Leeuw *et al.* (2017) encourage researchers to use storytelling ‘in ways that are open to evolution toward the unintended’ so that their transformational and decolonizing potential is not lost. Stories and narratives as a way to collect memories are methodologically similar and have been used for place-specific research with a historical component ( Feola *et al.*, 2023; Malhotra, 2016). It is important for researchers to apply methodological rigor and ethics when triangulating multiple (likely conflicting) narratives from a specific place and be transparent about which narratives or counternarratives are represented in published reports ( Redden *et al.*, 2022).

A focus on stories in our research therefore has the potential to achieve multiple goals that have been outlined in the preceding sections: 1) to contribute knowledge about the deeply contextual aspects of urban transformation and transformative capacity, specifically social learning and reflexive action (which likely occur through practices of storytelling) and 2) to support decolonization of knowledge about how specific cities have changed in ways that support equity, and in turn, 3) to contribute new narratives about how cities, in general, could facilitate processes of equitable and sustainable urban change.

## 3. Methodological approach

**Figure 1.**
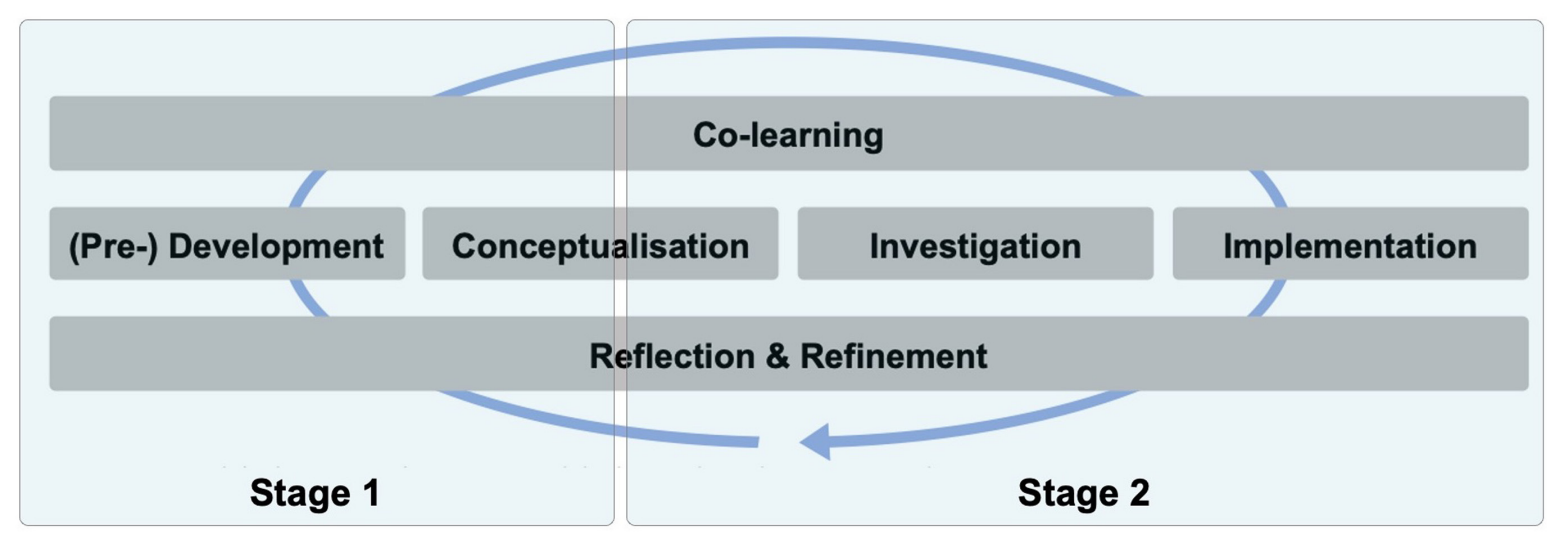
Stages of *Change Stories* research aligned to a new transdisciplinary research model ( Pineo *et al.*, 2021b).

We have adopted Pineo *et al.*’s (2021b) transdisciplinary research model ( Figure 1) to structure our research approach in a way that integrates diverse forms of knowledge and perspectives in project development and conceptualization. The model is formed of six inter-connected and non-linear phases. During stage 1 we gathered data from diverse sources, using participatory methods, to conceptualize the project (as described in this paper) and during stage 2 we will iterate and extend this work with new partners ( Figure 1). We worked over a nine-month period on the activities outlined in Table 2 in an iterative manner. We have broadly grouped these activities under expert input and literature review below. University College London Bartlett School of Environment, Energy and Resources Low Risk Ethics approval was received for the portion of this study involving U.S. interviews on 24 June 2022.

**Table 2.** Objectives and corresponding activities completed in Stage 1 of *Change Stories*.

Objective	Activities
1. Determine sustainable and equitable urban development knowledge gaps in U.S. cities	• Rapid literature review of current trends in sustainable and equitable urban development in the U.S. • Semi-structured interviews with 23 U.S.-based sustainable and equitable development experts. • Results of U.S. interviews inform site selection process.
2. Co-produce a conceptual approach and project structure for working across diverse geographies	• Rapid literature review of theories, frameworks, research approaches and methods. • Three workshops with advisory group to inform and iterate a draft research approach incorporating five principles as a conceptual framework.
3. Collaboratively select sites and partners to study narratives of equity-driven sustainable development	• Development and iteration of site selection criteria with advisory group (via online survey), and a participatory workshop. • Review of literature, websites and tacit knowledge of global experts (including advisory group) to identify 33 potential case study cities. • Explored suitability of potential sites with advisory group and a participatory workshop (~30 international participants). • Development of research partnerships with selected sites, using advisory group and research networks to identify collaborators.

### 3.1. Expert input

Using both formal and informal interviews and participatory workshops, we gathered input from a range of ‘experts’; a term that we use broadly in reference to individuals with lived experience, professional training or academic knowledge in the area of sustainable and equitable urban development ( Collins & Evans, 2002). Expert input was sought in multiple ways for different purposes ( Table 3) with international and U.S.-based individuals, aiming for geographic and sector diversity in both cases. We primarily used purposive and snowball sampling, contacting people on the basis of existing professional networks. When approaching academic experts, we sought out new perspectives by contacting the corresponding author of published studies (e.g. on decolonial methods). Discussions with academic experts were not recorded or analyzed, however notes were taken and summary points were discussed among the research team. Analysis of the semi-structured interview findings with U.S.-based participants involved hybrid (inductive and deductive) coding of transcripts related to our research questions (using Nvivo qualitative data analysis software). Thematic analysis was used and the process was iterative, involving presentation of summary findings to advisory group members to inform ongoing analysis. This paper reports headlines from the interview data, combined with literature, demonstrating how those results informed the context and study design for stage 2 of our study.

**Table 3.** Overview of the purpose and type of expert input, including: methods, expertise type, location and number of experts.

Method	Expertise and organization type	Location of experts	Number of experts	Purpose (and corresponding research objective)
Informal virtual meetings	Academic expertise in sustainable development	Australia, Brazil, Colombia, Canada, India, Nigeria, Rwanda, UK, USA, Zimbabwe	27	Identification of case study cities, potential partners and suitable research approaches. (Objectives 2 & 3)
Online workshops and surveys (Advisory Group)	Local government, academic, philanthropy, non-profit, consultancy, knowledge dissemination, health and sustainable development	Australia, Brazil, Canada, Colombia, Philippines, South Africa, Spain, Switzerland, UK, USA	14	To inform the study design, to build working relationships between a range of potential partners and to provide a learning opportunity for the project team and members. (Objectives 2 & 3)
Participatory workshop	Same organization types as above, additional expertise in housing, community gardening and other sectors	Same as above for advisory group participants, location of additional participants not recorded	Approx. 30	To inform potential case study cities. (Objective 3)
Semi-structured interviews	Local government, philanthropy, private sector, non-profit (included professionals and volunteers)	All in the USA (California, Florida, Kentucky, Massachusetts, New York, North Carolina, Ohio, Oregon, Pennsylvania, Tennessee, Washington)	23	To understand context, knowledge and practice about U.S. sustainable and equitable urban development. (Objective 1)

The advisory group was a point of collaboration with another RWJF grantee, the International Society for Urban Health (ISUH), that was concurrently running a project aligned with *Change Stories* called Accelerating City Equity (ACE) ( Dsouza *et al.*, 2023). Members of ACE and the *Change Stories* advisory group were convened for a participatory workshop (at the International Conference on Urban Health, in Valencia, Spain, October 2022). Approximately 30 participants contributed to refining the case study selections via this workshop.

### 3.2. Literature review

We conducted rapid literature reviews across multiple fields and topics throughout the course of the stage 1 study. Using the University College London library databased, peer-reviewed journal articles and scholarly books were reviewed to understand: 1) current trends in sustainable and equitable urban development in the U.S.; 2) theories and frameworks for sustainable and equitable urban development, and transformative urban change; 3) storytelling and policy-mobility; 4) case study, ethnographic and storytelling research methods; and 5) decolonial research approaches. We shared summaries of our findings with the advisory group and we identified scholars via their published research for expert interviews.

Potential case study sites were identified through grey literature, including published reports and websites, and academic studies. We reviewed reports and websites published by organizations such as Asian Coalition for Housing Rights, C40 Knowledge Hub, Centre for Sustainable Cities, Climate Smart Cities, Council on Urban Initiatives, Future Earth, Future Policy, Housing Europe, Housing Finance Africa, Institute for Transportation & Development Policy, Local 2030, Resilient Cities Network, World Bank, World Economic Forum, World Health Organization, World Resources Institute, Urban Nature Atlas, and UN Habitat. Only examples that met our initial selection criteria (see case study selection) were logged on to an online visual collaboration tool (Miro). We used these virtual cards (grouped by continent) to gather feedback from members of the team and the advisory group, directly in Miro (see Table 4). The initial selection criteria required sites to be working on health from a sustainability perspective and be centered on the goal of advancing equity.

**Table 4.** Potential cities that were screened for inclusion in the *Change Stories* project. *Indicates cities added by advisory group members.

No.	Location	Case study themes
	**Africa**	
1	Addis Ababa, Ethiopia	Healthy city planning, blue and green infrastructure, mobility
2	Jijna, Uganda	Community empowerment, renewable energy
3	Kampala, Uganda	Sanitation infrastructure
4	Kigali, Rwanda	Integrated resilience framework, incremental citizen centered urban planning, transport, low carbon development
5	Lagos, Nigeria	Resilience strategy
6	Cape Town, South Africa *	Upgrading informal settlements
7	(multiple), South Africa	Housing delivery for poverty alleviation, job creation and more
	**Asia and Oceania**	
8	Ahmedabad, India	Land for public purpose, waste management, waste picking initiative by non-state actors, heat action planning
9	Gaziantep, Turkey	Refugee integration
10	Honiara and Port Vila, Solomon Islands	Urban adaptation and climate resilience, community cohesion
11	Indore, India	Sustainable mobility, resource management, circular economy, community engagement
12	Jakarta, Indonesia	Urban Poor Network, community empowerment and support, affordable housing provision
13	Makassar, Indonesia	Livable City Plan, resilience, climate adaptation
14	Mandaue City, Philippines	Settlement upgrading, housing, climate adaptation
15	Nakhon Sawan, Thailand	Housing production for low-income groups and community engagement
16	Sydney, Australia *	Cool streets initiative empowering communities to take action on urban heat and carbon emissions through tree planting
17	Udon Thani, Thailand	Infrastructure development, waste to energy plant, mobility and transport innovations for walking and cycling
18	Yogyakarta, Indonesia	Community mobilization, development strategies through grassroots capacity, participatory methods
	**Europe**	
19	Barcelona, Spain	Mission-oriented model of housing affordability, public space
20	Belfast, Northern Ireland	Major change in governance context since Good Fridy Agreement, participation and community-driven green infrastructure in low-income neighborhoods, Belfast Healthy Cities network, strong community advocacy from Participation and the Practice of Rights (PPR).
21	Bristol, England	Low carbon and smart energy infrastructure in Bristol Leap carbon neutrality by 2030 plan
22	Helsinki, Finland	Housing First model to end homelessness
23	Malmö, Sweden	Climate change adaptation, nature-based solutions, BiodiverCity and EcoCity Augustenborg projects
24	Utrecht, Netherlands	Urban resilience and citizens’ wellbeing via green and blue infrastructure
	**South America**	
25	Belo Horizonte, Brazil	Urban agriculture, food security, participatory budgeting
26	Bogotá, Colombia	Care system for unpaid female caregivers, cable car transport, 16 projects of urban development for the poorest populations in informal settlements, Ciclovía Recreativa (streets closed to vehicles)
27	Curitiba, Brazil	Transit oriented development, bus rapid transit network, waste management
28	Medellín, Colombia	Governance, public space, education, mobility, urban identity, and ecosystem recovery
29	Porto Alegra, Brazil	Participatory budgeting, urban infrastructure, planning process
30	Quilombola, Brazil *	Self-funded family projects (Adan and Toninho), decolonial interventions
31	Rio de Janeiro, Brazil *	Revolusolar, solar energy project in the favelas of Babilônia and Chapéu Mangueira
32	Salvador, Brazil	Resilience strategy
33	Sao Paulo, Brazil	AgroFavela, a project to stop hunger, urban agriculture and food systems, empowering women

## 4. Situating the study for a U.S. audience

Literature review and semi-structured interviews provided our team with information about sustainable and equitable urban development in the U.S., informing our case study selection and plans for engagement with U.S. partners. In this section, we combine findings from both our interview data and literature review, and we report how each topic area informed our Stage 2 study designs ( Pineo, 2024).

### 4.1. Urban inequities facing U.S. cities

American cities face numerous environmental, economic and social challenges exacerbated by inequitable distribution of resources and procedural inequities, including disinvestment resulting in inadequate public infrastructure, disjointed governance models that marginalize community members from participating in decisions that affect them, and outdated or ineffective land development codes that undervalue the connection between people and their environments, and the contribution of urban development to environmental sustainability. Several participants described how segregation and exclusion are built into urban form and systems, separating people based on income, race and ethnicity, through policies and actions at multiple levels. Such observations have been made by American scholars for decades ( Acevedo-Garcia *et al.*, 2003; Massey & Denton, 1993; Trounstine, 2018; Wilson, 2012). There was a sense among interview participants that pointing out segregation and exclusivity is necessary due to lack of consensus on the matter and the need to provide local understanding of the history of structural racism in urban development by policymakers and the public. New data confirms old diagnostics. People of different social classes and races not only reside apart, but they move through different parts of the city ( Candipan *et al.*, 2021). They live lives apart. 

The historic practice of redlining was described as a causal factor for current disparities between neighborhoods and population groups. Race disparities in homeownership rates were one example: “it’s very, very, very obvious to all of us that the problem stems from redlining”. One participant said that American cities are “designed for exclusivity”, although a separate story indicated that some urban planners have only retrospectively understood their contribution to inequities: “frankly our down-zoning perpetuated that redlining. (…) We didn’t realize it”. Participants described various disparities in their cities that are driven by race, ethnicity and income, such as the quality of street maintenance or food access. Their observations are aligned with recent studies of U.S. cities (building on the foundation of environmental justice scholarship) connecting urban planning decisions with inequities across many outcomes, including: homeownership rates ( Sander *et al.*, 2018), life expectancy ( Huang & Sehgal, 2022), overheating ( Hoffman *et al.*, 2020; Hsu *et al.*, 2021), air pollution ( Lane *et al.*, 2022); noise ( Casey *et al.*, 2017) and many others.

### 4.2. Opposition to development and changing mindsets

In their work to improve equity in specific sectors and places, participants described encountering specific values and narratives that were underlying opposition to equitable development actions. Examples included racism and xenophobia, alongside lack of understanding among community members of how zoning regulations may change housing supply. For instance, one planner spoke of engagement with residents about proposed increases to density, stating ‘“we were hearing so much about ‘upzoning is the antithesis of home ownership, you are robbing us of home ownership’”. Another participant who described underlying racist and classist motives behind opposition to affordable housing said that there was little value in naming those motives because that makes people very angry, instead dialogue and relationship building is needed. Racist and classist motives for opposition to development policies and decisions have been identified by planning scholars ( Tighe, 2012). In addition, the FrameWorks Institute ( 2020) outlines ‘widely shared patterns of thinking in American culture that obstruct progressive change’, including individualism and stereotypes about marginalized groups. These observations underpin scholars’ concerns about the limits of communicative planning models, noting that opposition will undermine equitable outcomes for marginalized groups with weak political power ( Fainstein, 2000; Scally & Tighe, 2015) .

In addition to relationships and dialogue, participants described specific strategies for persuading people with opposing views. They explained how they “framed” proposals to win political or organizational support, communicated “narratives” to change mindsets and encouraged community members to tell “stories” to convince decision-makers of their position. The way that some participants who worked in local government spoke about their projects revealed carefully crafted narratives to explain equity and the need to target resources in communities that had been chronically disinvested. These findings resonate with claims that dialogue about urban issues occurs through storytelling (e.g. Innes, 2004; Sandercock, 2003) and that narrative is core to persuasion ( Fisher, 1989; McBeth *et al.*, 2022). The idea of deploying narratives to change mindsets has experienced a ‘recent swell’ among advocates, activists and some funders in the U.S., however there is a lack of knowledge about the circumstances and mechanisms through which such a strategy will be most successful ( FrameWorks Institute, 2020).

### 4.3. Building trust and capacity

Bringing forward equitable development policies and programs was described as challenging in the context of segregation, years of disinvestment and top-down development that created harms for low-income and racial minority communities. Trust was seen as a fundamental component to dialogue and collaboration, both of which were needed for multi-sector sustainable and equitable urban development. There are multiple components to the importance of trust as explained by participants. First, past harms have created distrust that has resulted in opposition and diminished collaboration. One participant said, “…communities have been planned to death. They’ve been over-promised. And because of that, there’s a massive trust deficit”. Second, trust is core to authentic dialogue and communication, which is necessary to change mindsets and reach agreement about problems and solutions. This point is exemplified by the follow quote:

“municipal staff and or elected officials need to talk to residents of color or low-income residents or residents that disproportionately face barriers to good health… In order for those conversation to move the dial, the residents need to trust the elected officials or the staff”.

Finally, trust was created through relationships, and both are needed for multi-sector working. The importance of trust in communication, planning and sustainable and equitable development activities has been well-documented ( Rydin, 2011; Stein & Harper, 2003). Yet the extent to which trust is a pre-requisite for such activities (Innes and Booher ( 2010) claimed it is not), and how it can be built or maintained in the context of conflict, are less clear ( Laurian, 2009).

In addition to trust being core to equitable development processes, participants also described the importance of efforts to increase their own capacity to work in this area. Doing work that pushes against the status quo was seen as difficult by many participants. Some participants spoke of how they gave or received support in terms of training, building networks and coalitions and providing space for healing. Multiple types of organizations were providing support to different actors, giving a sense of there being an ecosystem of capacity building across local government, professional groups and non-profits. Similarly, networks and coalitions were formed by different types of organizations to provide mutual support, enable dialogue and form collaborations. Support was required within and across organizations to reduce silo working and enable multi-sector approaches, which involved people working outside of their comfort zone.

### 4.4. Communities leading U.S. equity initiatives

Sustainable and equitable development initiatives were described as being most commonly led by communities, non-profits and government agencies working at different levels, however the bottom-up initiatives were described as offering the greatest potential for disrupting the status quo to achieve transformative results. Such efforts spanned a spectrum from activist grassroots efforts to “fight” for recognition and resources through to non-profit organizations that partnered closely with local government. It was notable in the interviews that some non-profit and community-based interview participants simultaneously acknowledged the importance of partnership with local government, while also noting their relative lack of capacity to progress innovative policies and programs. For example, one participant spoke of the difficulty in setting up a community land trust, “the amount of conversations that we had with the department of housing… with city council members, it was just Groundhog day… I don’t think was prepared for how much time it took to talk to city leaders”.

Bottom-up equitable development initiatives, whether by non-profits or committed residents, were described as necessary in the context of past failures to improve urban problems via top-down urban renewal programs (see Zipp & Carriere, 2013). There are numerous examples of successful community-driven initiatives in the USA, such as Dudley Street Neighborhood Initiative ( Agyeman, 2013). However, there is also a problematic history of opposition from local officials and poorly executed federal programs ( Green & Haines, 2012). The interviews demonstrated participants’ perceptions that undoing status quo (inequitable) development required at least procedural justice if not a more radical transfer of power and/or resources to communities for decision-making.

### 4.5. Integration of U.S. context in study design

Our semi-structured interview findings identified potential commonalities between U.S. and international case study cities. Such commonalities could be related to 1) social problems, such as the relationship between racism, discrimination and urban planning, 2) policy issues, such as lack of affordable housing and poor access to food, and 3) models of civic engagement, for example, participatory budgeting. With the advisory group, we discussed concerns about the perceived applicability of international urban change models for U.S. audiences because some research participants explicitly described how they viewed the USA to be different with respect to acceptable policy solutions (e.g. social housing is not a widely adopted model for affordable housing delivery compared to European countries). One implication from the interviews is that ‘transferring’ learnings about governance models (e.g. co-production and participatory governance) could potentially produce even greater insights than specific policy solutions.

## 5. Case study selection

Selecting the sites for the *Change Stories* ethnographically approached research (Stage 2) was an iterative process involving a balance between satisfaction of selection criteria and identification of research partners. The first four selection criteria were provided in the original grant brief from the funder, as follows:

1. First and foremost, all sites must be working on health from a sustainability perspective and be centered on the goal of advancing equity.
2. Sites should be urban but can vary in population or geographic size.
3. Sites should vary in terms of their geographic locations.
4. Researchers should select sites where they, or members of their team, are able to conduct deep ethnographic research.

An additional set of potential criteria were discussed with advisory group members, noting the difficulty of finding projects where all criteria could possibly be achieved. Table 5 shows the results of an online survey of advisory group members’ preferences for criteria. In conversations and the survey results, comparability across cases was considered problematic, but also desirable. It was unclear on what basis comparability should be evaluated (e.g. size, governance model, etc.). The ‘representativeness’ criterion (meaning representative of a U.S. process or context) was also discussed in-depth. As the stage 2 study results should inform a U.S. audience, there was a desire to match their needs and interests, yet some members of the project team and advisory group believed that the study’s focus on narratives and mindsets may produce results of relevance to any setting. Based on advisory group discussions and the U.S. interviews, we adopted a further four criteria:

5. Sites should have experienced a holistic, system-wide and multi-sectoral change, rather than a sector-specific change.
6. Sites should be representative of a process or context that is relevant to a U.S. audience (as informed by interviews and literature review).
7. Sites should demonstrate change in multiple parts of a city, not a single district or neighborhood.
8. Programs/policies/practices at the site should exemplify genuine participatory approaches.

**Table 5.** Advisory group's preferred ranking of selection criteria (n=7).

Criteria	Total	Ranking (based on total)	Mode
**Type of change:** Change is holistic, system-wide rather than a sector-specific or single place-based intervention.	17	1	1
**‘Representativeness’:** Sites are representative of a process or context that is relevant to the needs of a U.S. audience.	19	2	1
**Scale of change:** Sites demonstrate city-wide rather than a more local level (district or neighborhood) change.	25	3	3
**Comparability:** Sites have sufficient similarities (such as scale or type of change) to allow for comparison and synthesis of common lessons.	26	4	2
**Connected:** Researchers are suitably connected to each site to allow data collection with multiple types of stakeholders.	27	5	3
**Documented:** Sufficient existing documentation (e.g. for context, longitudinal data, etc.).	33	6	6

Using Miro for collaboration, a virtual card was created for 29 potential case study cities with a project summary and hyperlinks to further resources, with a further four examples provided by advisory group members ( Table 4). Five cities were then discussed at a participatory workshop in Valencia, including: Ahmedabad, Barcelona, Belo Horizonte, Bogotá and Kampala. Small groups with people who had experience of each city discussed their fit with the project selection criteria. Following these discussions, and consideration of other selection criteria such as potential local partners, the cities of Ahmedabad, Barcelona and Kampala were excluded. These decisions were difficult to take and reflected an overall balance between practical factors (e.g. local partners and anticipated costs) and alignment with the selection criteria. Barcelona was considered a sector-specific initiative (housing), while participants questioned whether Ahmedabad and Kampala had achieved community-engaged city-wide change that benefited the most vulnerable residents. Following further discussion in the project team and efforts to find local partners, the final three case studies for stage 2 were selected as Belfast, Belo Horizonte and Bogotá (see further detail below). With partners in each case study city, we developed an overarching research focus expressed as a broad research question (stated below) in the proposal for stage 2 funding.

### 5.1. Belfast, Northern Ireland

In the 1980s, Belfast shared many of the problems seen in British de-industrialized cities – lack of economic opportunity, crime and poverty. Simultaneously the city was badly damaged by The Troubles, an ethno-nationalist conflict, which saw the city physically divided according to ethno-religious identities, with conflict interfaces predominately in low-income neighborhoods. The Good Friday Agreement (1998) effectively ended violence and reduced residential segregation, but neighborhood-level inequities continued, leading some to call Belfast a ‘twin-speed city’ ( Murtagh, 2017). Residents had a sense that local and central government were not effectively solving their problems and thus multiple strong and active community organizations sought to fill that gap. One example has been Participation and the Practice of Rights (PPR) a local, non-sectarian non-governmental organization (NGO) that has led a range of economic, social and environmental justice campaigns, including ‘Take Back the City’ that has highlighted the lack of social housing in the city and the implications of growing income disparities. Other communities across the city have led major environmental regeneration schemes, such as the Connsway Community Gateway (CCG), which has combined physical infrastructure renewal (parkland, watercourses, footpaths/cycleways and a linear greenway) with community participation and programs. Passing through seven of the top 25% most deprived wards in Northern Ireland, the Greenway has been credited with bringing about dramatic change for residents’ health and wellbeing. Community-led initiatives, such as those led by PPR and the CCG have affected the city council’s approach to strategic planning. *We will ask how, in the context of recent sectarianism and segregation, have communities in Belfast been successful at bringing about significant change for marginalized residents, despite low input from government.*

### 5.2. Belo Horizonte, Brazil

Belo Horizonte has gained global recognition for its success in food security, climate change adaptation and mitigation measures, participatory budgeting and revitalization of informal settlements through innovative solutions involving community engagement and decision-making processes. These policies were defined as part of broader and more structural participatory processes that not only contributed to the definition of the policy objectives but also to their development and sustainability over time. This is exemplified by food security policies in the city. The early 1990s was a time of social mobilization against hunger and poverty in Brazil, leading to the election of the Partido dos Trabalhadores (Workers’ Party) in Belo Horizonte. High prices and poor access to food were causing food insecurity for Belo Horizonte’s approximately two million citizens. The city is now described as ‘a world pioneer in governance for food security’ after the 1993 Municipal Law established a commitment to the concept of food sovereignty: ‘the right of peoples to define their own food and agricultural policies, to protect and regulate their production and trade in such a manner as to secure sustainable development, to determine the degree of their autonomy and to eliminate dumping on their markets’ ( FuturePolicy.org, 2017). A social movement for ethics in politics mobilized up to 30 million people. Another example is the community participation in the Vila Viva urban renewal program, which is credited with significantly improved outcomes for residents, including reduced homicide rates and improved access to utilities ( Carvalho *et al.*, 2012). *We will ask how Belo Horizonte has maintained equitable development practices for decades, particularly in the context of significant economic and political changes.*

### 5.3. Bogotá, Colombia

Bogotá, Colombia’s capital, is a vibrant city of more than 8 million people and a deeply unequal and fragmented city ( Aliaga-Linares & Álvarez-Rivadulla, 2010). In recent years, however, the city has innovated a series of urban policy and planning initiatives aimed at fostering inclusion and sustainability. Bogotá boasts one of the globe's largest Bus Rapid Transit (BRT) networks and Ciclovía, the world's largest event dedicated to promoting cycling and walking, facilitating community engagement and sustainable mobility practices ( Montero, 2020). And while some of these initiatives have garnered global recognition, positioning Bogotá as a model for progressive planners and advocates worldwide, some transport infrastructure and urban redevelopment projects have also been highly controversial. This shows that the simplified story that circulates globally about Bogotá does not always reflect the complexity of what happened on the ground ( Montero & Baiocchi, 2021). A recent equitable urban development shift has occurred in the city, centered around the needs of unpaid female caregivers. In Bogotá, 88% of girls and women engage in unpaid care work and 27% of them spend an average of 6 or more hours per day doing unpaid care work. Low socioeconomic neighborhoods have the highest concentration of unpaid caregivers. A new care system initiative, called *Manzanas del Cuidado* (Care Blocks), aims to give women more time and autonomy by enabling the equitable distribution of unpaid domestic care work across genders. The program involves both reorganization of new and existing physical infrastructure and the provision of clustered services, distributed across the city and managed by integrated institutions from multiple sectors. *We will ask how, in a patriarchal culture, did the needs of unpaid female caregivers receive such prioritization for resources*.

## 6. Stage 2 research approach

The research development activities reported above led us to develop the following vision and aims for the primary *Change Stories* study (stage 2). Our vision for the *Change Stories* project is to **shift the paradigm of how we tell stories about successful urban change and who tells them, leading cities to develop deeply contextual solutions to support health, equity and wellbeing**. This project aims to understand the narratives and mindsets that have underpinned capacity for sustainable equitable development in three non-U.S. cities. With participants’ permission and collaboration, we will share each city’s “change stories”, with support from skilled storytellers. Through intentional communication efforts, we hope that the stories told through this project will open new possibilities, dialogues and perspectives for the problems facing cities in the U.S. and globally. By adopting equitable and de-colonial research approaches and reflexive practices, we will contribute methodological and conceptual innovations about epistemological pluralism.

### 6.1. Conceptual framework

Our project conceptualization has focused on collaborating and conducting research across diverse knowledges and geographies. We produced a set of principles that inform the way that we will work together to create and share knowledge in stage 2. These principles are an output of the stage 1 activities reported above (including interviews, literature review and advisory group discussions). We intend to further iterate these principles with our community partners.

***6.1.1. Equity and reflexivity.*** Our project is led and delivered in a culture that acknowledges (in)justice and continually works to address imbalances of privilege and power. Adopting decolonial research approaches involves ongoing reflection and iterative processes that take into account diverse knowledges. Furthermore, reflexivity in studies working across diverse disciplines and partnerships is important ( Pineo *et al.*, 2021b). Multi-country research partners must respond to real and perceived power dynamics and equity within the project, which also supports ethical integrity in research ( ESSENCE on Health Research and UKCDR, 2022). Projects should begin with the shared intention to create and maintain safe spaces for research partners to consider their own power and privilege, and respond to different positionalities among partners ( Maclean *et al.*, 2022).

We will use reflection and evaluation to refine our processes and to avoid practices that ‘(re)produce and entrench norms of ubiquitous white-centric, heteronormative, patriarchal colonialisms’ ( de Leeuw *et al.*, 2017, p. 154). This activity will take place through meetings of our research team (which includes community-based partners) and advisory group. In addition to ‘reflexive evaluation’ of our research design, data collection and data analysis, Nicholls ( 2009, p. 118) highlights the importance of ‘political and relational layers of reflexivity…to critically evaluate empowerment and participation in a counter-colonial context’. Nicholls refers to a multi-layered reflexivity, with at least three layers (self, inter-personal and collective). As we aim to understand participants’ stories, and in some cases share those stories, in our goal to create change in other places, we will need to be particularly focused on this layer of reflexivity. This will need to occur in each site where our research is conducted, through our own reflection and by speaking to participants. 

***6.1.2. Participation and transparency.*** As we value diverse forms of knowledge, our project prioritizes participatory knowledge building in its design and delivery with a range of partners and people (e.g. communities, government, researchers). Part of our participatory research approach constitutes ‘co-production’, which is described as ‘knowledge arising from collective dialogue among actors with different expertise’ ( Castán Broto *et al.*, 2022, p. 2). A co-production approach is often implicit in transdisciplinary studies ( Pineo *et al.*, 2021b) and is well-suited to our research, not least because of its potential to support epistemic justice in our case study cities ( Castán Broto *et al.*, 2022). However, there are risks associated with over-stating the extent to which all project partners are engaged in knowledge production, such as ‘black-boxing elements of the knowledge process that need closer scrutiny’ ( Bremer & Meisch, 2017, p. 12).

Transparency in our interactions with project partners will support our co-production aims (ibid). We will partner with community-based organizations (CBOs) in each case study city and through our advisory group. CBOs will be involved in data collection and interpretation. We will foster participation and co-production by transparently communicating the project’s goals and limitations and collaborators’ diverse perspectives, positionalities and priorities. Through our interactions in meetings and workshops, particularly with community-based partners, we will work to build trust and collaborative working relationships, in which we will expose and work through conflicts ( Fam & Sofoulis, 2017). We will adequately compensate research participants (i.e. incentive vouchers) and continually reflect upon how we can legitimate their knowledge in reporting ( Reyes Cruz, 2008). We will also use research meetings to reflect upon the ‘hyphen-spaces’ ( Fine, 1994) between ourselves and participants, specifically how we might ‘work the hyphen’ to surface identity relations and their implications ( Cunliffe & Karunanayake, 2013).

***6.1.3. Pluralism.*** We seek to decolonize knowledge and knowledge production by empowering local partners to determine appropriate questions, methods and meanings, and by collaborating to combine knowledge from each case study. Learning from other cultures requires the *Change Stories* project to understand and be able to work with different conceptualizations of knowledge and ways of knowing. Members of the project team may have difficulty recognizing and understanding the meaning of stories that we hear from different worldviews and cultures. Building relationships will support us to create a shared language and respect for diverse epistemologies ( Lytle, 2009). 

There are several specific ways that we will incorporate diverse perspectives in our study design and conduct. For instance, rather than pre-specifying sustainable development and urban transformation theories to inform our research questions and methods, our regional co-investigators will determine appropriate theoretical lenses for the cities in which they are working. When synthesizing learning across cities, we will gather and analyze data with the goal of identifying ‘repeated instances’ of how change has been supported across the case study cities, aiming for mid-level abstraction, building on the ‘a posteriori comparisons’ methodology ( Montero & Baiocchi, 2021).

***6.1.4. Multi-layered impact.*** We seek to create impact through connecting places, stories and people, and adopting creative ways to amplify and sustain new links. We conceptualize impact in our research in relation to our focus on urban equity, the multi-sited nature of our project and the flow of knowledge across academic boundaries. Decolonial research approaches can contribute to epistemic justice in ways that change material conditions to improve equity via processes termed ‘emancipatory circuits of knowledge’ ( Butcher *et al.*, 2022). These knowledge movement ‘circuits’ are considered emancipatory in cases ‘where they entail the intentional redistribution of resources and authority and seek to address multiple dimensions of inequality: *redistribution, recognition, participation,* and *solidarity and care*’ (ibid, p.209). In addition, our research impact goals align with Beckett *et al.*’s (2018) multi-layered social impact framework, combining notions of context and scale to aid with understanding the potential impacts from complex and uncertain research projects, specifically those involving co-production. In broad terms, their framework outlines micro, meso and macro impacts across individuals, groups, institutions and society, which can function through mechanisms such as new relationships, norms and policies. We aim for our project to create such impacts in each of the case study cities, among U.S. audiences (that we will specify) and internationally. Similar to Moore *et al.* ( 2021), we will collaboratively develop a project theory of change to guide our understanding and implementation plans to convert research activities into specific impacts.

### 6.2. Investigation and implementation

**Figure 2.**
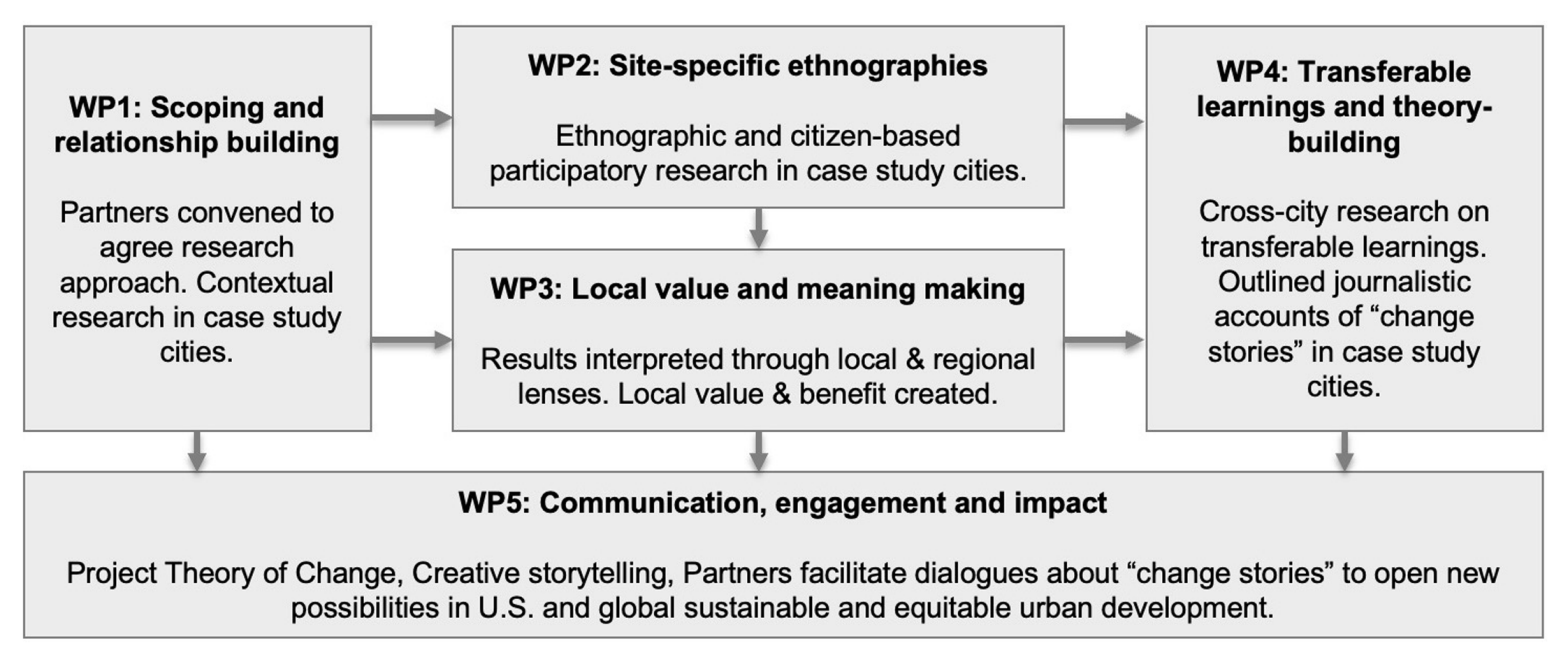
Flow of work packages (WP) in *Change Stories* stage 2, including description of core activities in each WP. Arrows indicate knowledge and information flows between work packages.

Our plan of work proceeds through five work packages (WP), ( Figure 2), which we describe in a summary format below.

Partnership working is core to the project and thus significant time is devoted to relationship building and establishing ways of working together in WP1. Case study cities will each review the historical, social, political, economic and cultural context of each city through desk-based research and 10-15 semi-structured interviews. As we develop partnerships and research ethics procedures, we will focus specifically on the ethics of storytelling ( Gubrium *et al.*, 2020) and community-based research in ways that reflect the importance of respect, beneficence, justice and ‘the ethics of relationships, care, and solidarity’ ( Fielding-Miller *et al.*, 2022). Our detailed research approach will be further established through a week-long residential knowledge exchange session (or retreat) with research partners, CBOs and key individuals in each city. The format is designed to provide tangible benefits to participants (networking, knowledge and skills) and researchers (data gathering, relationship building). We will share facilitation responsibilities and use participatory activities, such as storied introductions ( de Leeuw *et al.*, 2017), to learn about each other and our perspectives on the project. Our learning activities may further reinforce our capacity to produce transformational change in our respective locations, such as by enhancing leaderships skills and building relationships with other participants ( Wolfram, 2016).

Building on the scoping work above, the co-investigators will lead investigations according to the research questions and methods specific to their local context in WP2. Communication with research participants will use local languages and understandings of important topic areas, such as equity and sustainability. All cities will use some form of citizen-based participatory research (CBPR) in collaboration with community-based organizations. Methods may include storytelling interviews, guided walks, photo-voice, participatory video/digital storytelling and others ( Gubrium *et al.*, 2020; Harper *et al.*, 2012; Mistry *et al.*, 2014; Yap, 2022). Additional ethnographic research will be led by the co-investigators including interviews, document review, attending local events and site observations.

The focus of WP3 is to create local value from the research. Preliminary findings will be shared periodically with project partners and the advisory group to inform further investigation. Partners in each case study city will analyze qualitative data (from WP1&2) in the context of city and regional trends and challenges, alongside relevant urban development theory. In addition, partners will action opportunities for local benefits arising from the research, such as creating locally relevant training resources, to produce value for local actors and to avoid epistemic injustice.

We will develop transferable learnings and contribute to relevant sustainable and equitable urban development theories via WP4. Data will be gathered through meetings with project partners using methods such as storytelling. In virtual meetings, participants will speak in English or their native language (with automated sub-titles), to describe the stories and themes emerging from the research while also hearing and responding to other cities’ findings. Rather than translating the empirical ethnographic and other data from each city, we will rely on local partners’ interpretation and perspective of their emerging findings. This approach intentionally foregrounds local knowledge and prioritization of what is most important in each city. When analyzing this data, we will identify ‘repeated instances’ of how change has been supported across the case study cities using Montero and Baiocchi’s (2021) ‘a posteriori comparisons’ methodology. We will work with partners to develop short and journalistic “change story” outlines for each city, co-produced by local partners, which highlight the narratives and mindsets that have led to equity-driven sustainable development over time.

Beginning with the project inception, we will be developing relationships and formulating plans for communication, engagement and impact (WP5). We will begin by creating a Theory of Change, which will inform the development of an engagement strategy and plan. We will explore a variety of tactics for communicating stories with U.S. and global audiences (e.g. a podcast series, documentaries, storytelling roadshows) and will develop partnerships to co-produce materials. For reaching U.S. audiences, we envision that networks and intermediaries will be key partners to help us reach non-profits, urban development professionals, historically marginalized residents and local government officials. Through interactive storytelling exchanges between our global partner cities and others, we aim to facilitate an exchange of ideas to achieve multiple purposes. Our Theory of Change will elucidate the ways in which a storytelling process ‘unsettles Western mindsets/power and activates the political agency for transformative change’ ( Ortiz, 2022), while also opening possibilities out of crises ‘by engaging directly in collective and speculative imagination’ ( Harris, 2023). Although face-to-face events will be important for these exchanges ( Linde, 2001; Montero, 2017), we will also deploy virtual and digital storytelling modes to reach wider audiences. Photographs and audiovisual recordings taken through the project will be professional edited to creatively tell stories of change in each city. Multilingual videos will reach a wider audience and have the potential to influence global policy and decision-makers ( Montero, 2018).

## 7. Conclusion

The value of complex multi-country transdisciplinary research projects could be far greater to society if they were routinely given sufficient time for the crucial stages of development and conceptualization ( Pineo *et al.*, 2021b). This paper has reported a case where funding for a participatory research process was used for such project development, with significant benefits for the project team, the resulting project and hopefully its eventual contribution to policy and the health of the public. In this section, we outline reflections on our approach that may benefit future teams developing similar studies.

There are strengths and limitations to the methods we used in the development stage of *Change Stories.* Our participatory approach to designing a multi-country transdisciplinary study opened up the research process to a broader range of actors than would typically be involved in study design, increasing the potential rigor and impact of the resulting approach ( Funtowicz & Ravetz, 2015; Zimmerman *et al.*, 2016). We feel that, on balance, our case study selection process was a strength of our approach; however, we acknowledge that it involved a balancing act between research needs and practical considerations. Sites that otherwise met our selection criteria were not included due to lack of a suitable local partner (among other barriers). We likely considered a broader range of cities than would usually be possible in research proposal timelines. We pursued cases that would have afforded greater geographic diversity (e.g. in Asia and Africa), but the final number was limited by funding constraints. Our purposive sampling approach for research participants and advisors could be seen as a limitation. The experiences and expertise that we included were more aligned to our existing ways of thinking; however, we did encounter productive conflict and diversity of thought in expert input (particularly regarding decolonial research approaches). A final limitation in this development grant was the relatively late involvement of community-based partners in the co-production of our stage 2 research proposal. However, this can be overcome with their high involvement throughout the Stage 2 study. The selection of case study cities and partners was very late in stage 1, at which point the local academic partners felt that without certain funding for stage 2, it would be unfair to expect substantial contributions from CBOs. This could be a point of learning for the development phase of future transdisciplinary studies involving CBOs.

The risks and benefits of transdisciplinary research have been widely documented and we believe they merit further reflection because changes are needed within research institutions, funders and journals. Perhaps the principal benefit of transdisciplinary approaches is their potential to unsettle problematic status quo behaviors and develop solutions to complex societal problems. The relative lack of available funding for decolonial and transdisciplinary studies meant that the research team (both in stage 1 and stage 2) was excited about the potential for the *Change Stories* project to produce valuable knowledge and impact. There has been a tangible and (perhaps uncharacteristic) sense of positivity emerging from our belief that the power of individual and collective stories to drive substantial urban change could be a radical solution, capable of being scaled, inspiring hope and resolving problems. Likewise, the rigorous process in the development grant provided time and space to develop new partnerships and alliances to productively achieve these goals. We are hopeful that our transdisciplinary approach encourages and legitimizes a nested set of goals for the research (both locally-derived and collectively produced), which will support decolonizing knowledge production ( Evans *et al.*, 2020).

Some of the known risks of transdisciplinary research have also arisen in our project, but have been reduced through this development stage. As documented elsewhere, integrating disciplines inevitably produces some level of conflict about epistemological views that can be emotionally difficult for researchers ( Black *et al.*, 2019; Moore *et al.*, 2021; Pineo *et al.*, 2021b). We encountered diverse understandings of coloniality and decolonial research approaches, prompting collective and personal reflections on power and positionality within the project (see Eriksen, 2022), which were directly related to study design decisions. Benchmarks for ‘good science’ are intertwined in colonial and extractive research practices that de-value other ways of knowing ( Abimbola, 2019; Fam & Sofoulis, 2017), requiring researchers to take a stand against norms in their respective disciplines and institutions. We believe that this development grant has helped to reduce the level of conflict and risk that can arise when working across disciplinary and sector boundaries. By receiving funding to build new partnerships and collaboratively develop our research approach, we have made strong progress on understanding each other’s disciplinary perspectives and institutional requirements ( Lynch, 2006), enabling a strong start for integrative research in stage 2 of *Change Stories*.

In closing, we would like to highlight the importance of ongoing reflexivity in multi-country transdisciplinary research. Such projects navigate numerous ethical and methodological dilemmas that cannot be resolved through adherence to the positivist research protocols typical of health sciences. In adopting creative, ethnographic and community-based participatory research methods we will need to attend to ethical considerations that are not typically evaluated by institutional ethics committees ( Kara, 2020). Instead, researchers have a responsibility to consider the impact of their activities regularly and make adjustments in accordance with their values and professional expertise. For example, telling stories of urban change from an academic perspective involves balancing an accurate (or neutral) account of events with the subjectivity of participants’ stories. The research task of weaving different narratives together and triangulating information must show subtleties and conflict, while simultaneously paying attention to context and agency. Without careful attention to nuance and transparency in our process, the results may betray our informants in many ways. Our research team is committed to ongoing reflexivity to manage such challenges, but we do not expect these issues to be fully resolvable.

## Ethics and consent

University College London Bartlett School of Environment, Energy and Resources Low Risk Ethics approval was received for the portion of this study involving U.S. interviews on 24 June 2022. Participants were informed of the research topic and procedures and provided written consent prior to interviews.

## Data Availability

The case study selection process data are available as part of the article and no additional source data are required. The semi-structured interview data can be found as follows: Dryad: Semi-structured interviews about sustainable and equitable development in the USA. https://doi.org/10.5061/dryad.prr4xgxts ( Pineo, 2024). Data are available under the terms of the Creative Commons Zero "No rights reserved" data waiver (CC0 1.0 Public domain dedication).
