# Supplementary material for: Mobilizing knowledge about urban change for equity and sustainability: developing ‘Change Stories’, a multi-country transdisciplinary study
Source: Wellcome Open Res. 2024 Apr 24;9:218. [Version 1] doi: 10.12688/wellcomeopenres.21180.1 (PMC11364977; doi:10.12688/wellcomeopenres.21180.1)
Supplement: Peer review history

# Reviewer report: Reviewer response for version 1

**DOI:** 10.21956/wellcomeopenres.23425.r86989

**Published:** 2024-08-30

**Authors:** Nishita Dsouza

**Affiliations:** 1 Columbia University, New York, New York, USA

This paper showcases a research protocol for investigating and documenting urban change that is innovative, equitable, and non-extractive. The paper describes important concepts in depth, such as coloniality, social change at varying scales, and adapting lessons learned about transformation to different urban contexts. This paper is unique due to its focus on processes and detailed documentation of the participatory research methods for narrative storytelling of urban change. The paper is very well written and organized, with clear headings and figures.

Is the work clearly and accurately presented and does it cite the current literature?

Yes

If applicable, is the statistical analysis and its interpretation appropriate?

Not applicable

Are all the source data underlying the results available to ensure full reproducibility?

No source data required

Is the study design appropriate and is the work technically sound?

Yes

Are the conclusions drawn adequately supported by the results?

Yes

Are sufficient details of methods and analysis provided to allow replication by others?

Yes

Reviewer Expertise:

urban health, community engaged research

I confirm that I have read this submission and believe that I have an appropriate level of expertise to confirm that it is of an acceptable scientific standard.

# Reviewer report: Reviewer response for version 1

**DOI:** 10.21956/wellcomeopenres.23425.r82634

**Published:** 2024-06-20

**Authors:** Tisha Holmes

**Affiliations:** 1 Department of Urban & Regional Planning, Florida State University, Tallahassee, Florida, USA

This paper is well written, thorough, substantially grounded in the literature and presents compelling cases for conceptualizing and conducting the various stages of the decolonial global research on urban sustainability and equity. The authors go into great detail about the levels of commitment entailed in this type of work as well as potential limitations of their approach. The authors were attentive to communicating ideas using accessible language for the majority of the paper. The literature review was more complex and nuanced, but authors did well with walking the reader through these more theoretical concepts and their connections. I was hoping to see some commentary about the transdisciplinary make-up of the research team and the pre-work that they engage in individually and/or as a team to reflect on their positionality as they prepare to embark on the second phase of the project.

Is the work clearly and accurately presented and does it cite the current literature?

Yes

If applicable, is the statistical analysis and its interpretation appropriate?

Not applicable

Are all the source data underlying the results available to ensure full reproducibility?

Yes

Is the study design appropriate and is the work technically sound?

Yes

Are the conclusions drawn adequately supported by the results?

Yes

Are sufficient details of methods and analysis provided to allow replication by others?

Yes

Reviewer Expertise:

Climate resiliency planning; Community based planning

I confirm that I have read this submission and believe that I have an appropriate level of expertise to confirm that it is of an acceptable scientific standard.
